# Association Between Mesh Placement and Recurrence and Chronic Pain After Incisional Hernia Repair: A Systematic Review and Network Meta‐Analysis

**DOI:** 10.1002/wjs.70390

**Published:** 2026-05-06

**Authors:** Camilla Witthøft, Usamah Ahmed, Evy Á Lakjuni Guttesen, Jacob Rosenberg, Jason Joe Baker

**Affiliations:** ^1^ Center for Perioperative Optimization, Department of Surgery Copenhagen University Hospital—Herlev and Gentofte Herlev Denmark

**Keywords:** chronic pain, incisional hernia, recurrence, surgical technique, ventral hernia

## Abstract

**Background:**

Recurrence and chronic pain remain significant challenges in incisional hernia repair, and evidence on the optimal mesh placement is limited. This review aimed to determine whether there is a difference in the risk of recurrence and chronic pain based on mesh placement in patients undergoing incisional hernia repair.

**Methods:**

Studies with adults undergoing elective incisional hernia repair for defects < 10 cm were included. PubMed, Embase Ovid, and Cochrane CENTRAL were searched on August 18, 2025. RCTs were assessed with Cochrane's Risk of Bias tool, version 2. Cohort studies were evaluated with Cochrane's Risk Of Bias In Non‐randomized Studies of Interventions, version 2. Meta‐analyses and a network meta‐analysis were conducted to compare recurrence rates across placements. The protocol was pre‐registered in PROSPERO (CRD420251148033).

**Results:**

Twenty‐two studies with 10,832 patients were included. Crude recurrence rates were highest for preperitoneal (12.8%) and lowest for retromuscular (3.0%) mesh positions. In the network meta‐analysis, retromuscular (RR 0.3, 95% CI 0.1–0.8) and intraperitoneal (RR 0.4, 95% CI 0.2–0.9) placements were significantly associated with a lower risk of recurrence compared with onlay. However, the certainty of evidence was very low due to high risk of bias and heterogeneity, limiting confidence in these estimates. Four studies reported chronic pain, but substantial heterogeneity precluded meta‐analysis.

**Conclusion:**

Retromuscular mesh placement may reduce recurrence compared with onlay mesh. However, these findings were limited by clinical and statistical heterogeneity across studies. Reports on chronic pain were few and heterogeneous, needing further research on the link between chronic pain and mesh placement.

## Graphical abstract

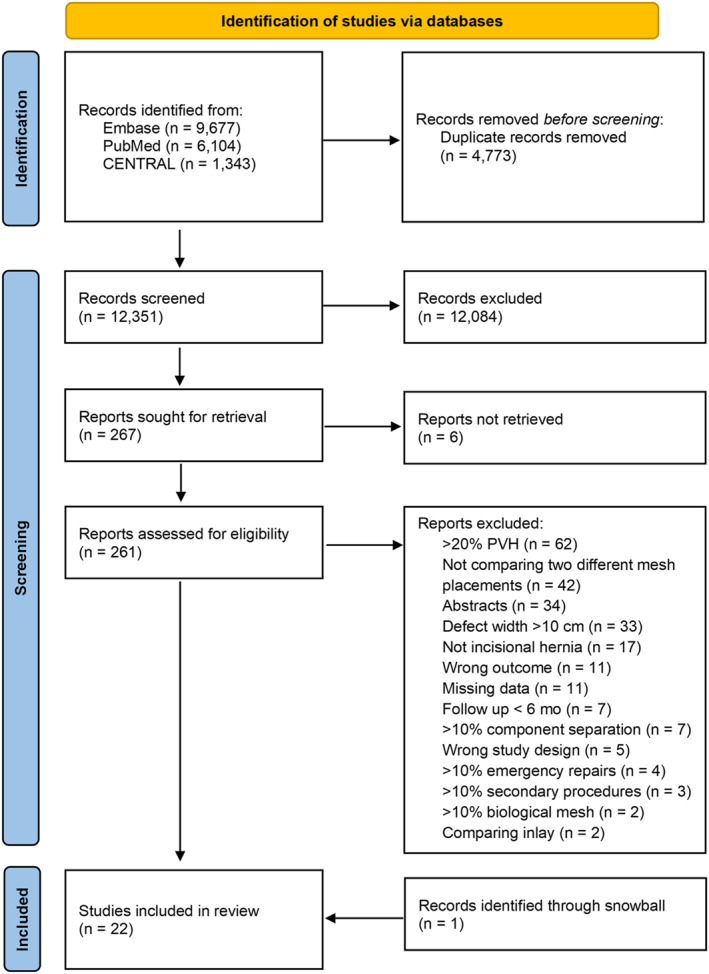

## 1 Introduction

Incisional hernia repair remains a clinical challenge due to its high complication rates. Compared to patients undergoing primary ventral hernia repair, those with incisional hernias face higher rates of recurrence, readmissions, and wound complications [1, 2]. Nevertheless, only a limited number of studies have specifically examined incisional hernias, affecting how we evaluate treatment outcomes and their prevalence [3]. Recurrence rates tend to be high for patients with incisional hernias, but they fluctuate considerably across studies, with up to 2/3 of patients experiencing recurrence [4, 5].

The use of mesh is the standard approach for ventral hernia repair [6], with various anatomical placements available. It can be positioned as onlay, retromuscular, preperitoneal, or intraperitoneal (IPOM) [7]. Over the past decade, there has been a decline in the use of IPOM, while extraperitoneal placements have gained popularity among surgeons [8]. The European Hernia Society [6] currently recommends retromuscular mesh placement for incisional hernia repair; however, they acknowledge that the literature is heterogeneous, and that the quality of the evidence is insufficient to make a firm recommendation. Therefore, the true impact of mesh placements on recurrence and chronic pain remains unclear. The literature also reports high rates of chronic pain after incisional hernia repair, ranging from 15% to 27% [9, 10]. However, the extent to which mesh placement contributes to the development of chronic pain remains uncertain. These complications have a considerable negative impact on patients' quality of life, and up to 23% of patients remain dissatisfied after surgery [5].

The aim of this systematic review was to determine whether there is a difference in the risk of recurrence and chronic pain associated with mesh placements in adults undergoing incisional hernia repair.

## 2 Methods

### 2.1 Study Design

This systematic review with network meta‐analysis was reported following the PRISMA‐NMA guideline [11]. The protocol was pre‐registered in PROSPERO with registration number CRD420251148033 [12].

### 2.2 Eligibility Criteria

This review included studies with adults (≥ 18 years old) who underwent elective incisional hernia repair with defect widths < 10 cm. If 1 group in a study had a mean defect width ≥ 10 cm, the study would be included if the overall mean defect width for the entire cohort was < 10 cm. The studies had to compare at least 2 different mesh placements, either onlay, retromuscular, preperitoneal, or IPOM [7]. The outcome had to be recurrence or chronic pain, and follow‐up should be at least 6 months after surgery. The review included randomized controlled trials (RCTs) and cohort studies. Studies were excluded if they pooled primary ventral and incisional hernias, where primary ventral hernias accounted for more than 20% of the cases. We also excluded studies with more than 10% of either resorbable, biological, 3‐dimensional mesh, Physiomesh, Ethicon, New Jersey, emergency repairs, component separations, or contaminated field.

### 2.3 Information Sources

The search was conducted in PubMed from 1966, Embase Ovid from 1974, and Cochrane CENTRAL, which was searched on August 18, 2025. Forward and backward citation searches [13] were conducted on all studies included, using both Google Scholar and PubMed. The search strategy was developed in collaboration with an information specialist. There were no language restrictions, but the search was only conducted in English. The full search strategy in PubMed was: ((“ventral” OR “incision*” OR “abdominal”) AND hernia) OR “Hernia, Ventral”[Mesh]) AND (onlay OR premuscular OR sublay OR retromuscular OR retrorectus OR preperitoneal OR intraperitoneal OR IPOM* OR underlay OR open OR robot* OR laparoscop* OR “Laparoscopes”[Mesh]) AND ((“Recurrence”[Mesh] OR “Reoperation”[Mesh] OR Recurrence* OR Reocurrence OR Reoperation OR Re‐operation OR Relapse*) OR (pain OR “Pain”[Mesh] OR “prom” OR “proms” OR “pro” OR “pros” OR “Patient‐report*” OR “Patient report*” OR “self‐report*” OR “self report*” OR “QOL” OR “Quality of Life” OR “Quality of Life”[Mesh] OR “Patient Reported Outcome Measures”[Mesh] OR “Self Report”[Mesh])). No filters were applied. The search strategy was adapted to Embase Ovid and Cochrane CENTRAL and is available in the PROSPERO protocol [12].

### 2.4 Selection Process

Covidence [14] was used to manage the screening process and automatically removed duplicates. Title and abstract screening were conducted independently by 2 authors, and any disagreements were resolved through discussion. Full‐text screening was conducted by 1 author in collaboration with another author. In case of missing data or uncertainty about inclusion criteria, the study's corresponding author was contacted by e‐mail to determine if the study could be included. Studies reporting duplicate populations were excluded from analyses, ensuring that each study population was included only once.

### 2.5 Data Collection Process

The data were extracted by two independent authors using a pre‐piloted data extraction form. If there were any ambiguities or uncertainties, it was discussed with a third author. Data were extracted on study characteristics (design, year of publication, authors, follow‐up), patient characteristics (age, sex, hernia defect size, hernia type, comorbidities), and operative details (mesh placement, type of mesh and fixation, including if IPOM was with defect closure (IPOM+)) [15]. Defect size was reported as diameter (width). In cases where the original study presented defect size as an area, the value was recalculated to diameter using the formula: $A=\pi \cdot r^{2}$. All other data were presented as the included studies presented them. No data imputation was performed for missing data.

### 2.6 Study Risk of Bias Assessment

Each study was assessed for bias by 2 authors, and a third author was consulted if any disagreements arose. RCTs were assessed by the Cochrane Risk of Bias 2 (RoB 2) tool, with the following domains: bias due to the randomization process, deviations from intended interventions, missing outcome data, measurement of outcomes, and selection of reported results [16]. Cohort studies were assessed with Risk Of Bias In Non‐Randomized Studies—of Interventions (ROBINS‐I V2), which evaluated several domains: bias due to confounding, participant selection, intervention classifications, deviations from intended interventions, missing data, measurement of outcomes, and selection of the reported results [17]. Publication bias could not be assessed, since there were fewer than 10 studies in each meta‐analysis [18].

### 2.7 Effect Measures

For recurrence, crude rates were derived from individual studies, while pooled risk ratios were derived from meta‐analyses and network meta‐analysis. For chronic pain, data were presented as reported in the included studies and summarized narratively.

### 2.8 Synthesis Methods

Meta‐analyses and network meta‐analysis were performed using MetaXL [19]. Network meta‐analysis was conducted using the Generalized Pairwise Modeling (GPM) framework implemented in MetaXL. This method constructs all possible direct and adjusted indirect comparisons within closed loops, assuming transitivity. Indirect estimates were generated using the Bucher method, and both direct and indirect effects were subsequently synthesized through standard pairwise meta‐analysis. Variances for indirect estimates were calculated as the sum of the variances of the contributing direct comparisons. Consistency assessment followed the principles inherent to the GPM approach. Internal coherence was evaluated by comparing direct and adjusted indirect estimates within each closed loop. As MetaXL does not include separate node‐splitting or design–by–treatment inconsistency models, no additional statistical inconsistency tests were applied beyond these internal checks. Bayesian modeling and multivariate variance structures were not used, as they are not part of the MetaXL framework. Random effect model was applied due to methodological and clinical heterogeneity among studies, and statistical heterogeneity was assessed using I^2^ statistics [20].

IPOM placement included intraperitoneal mesh, both with and without defect closure. The effects of treatments were expressed as risk ratios. Onlay mesh placement was used as the reference group, as it was the most frequent comparison group, along with IPOM. If zero events occurred in a group, MetaXL automatically applied the continuity correction principle that adds 0.5 “events” to both the group with zero events and the group it is being compared to. This prevents division by zero as risk ratio would not be possible to calculate otherwise.

Sensitivity analyses were conducted to assess the robustness of the results, first excluding studies relying solely on reoperations as a proxy for recurrence, because using reoperation alone fails to capture patients with recurrence who are not reoperated, thereby underestimating the true recurrence rate [21]. Another sensitivity analysis excluded studies with follow‐up periods of less than 24 months, as previous research has shown that recurrence rates continue to increase over time [1]. Additionally, a sensitivity analysis was conducted separating studies using IPOM with and without defect closure, as evidence suggests that IPOM+ is associated with lower recurrence rates [22]. We conducted one analysis including only studies with clinical examination for recurrence at follow‐up. Finally, we also performed sensitivity analyses excluding all studies with an overall high risk of bias, including only RCTs, and including only RCTs without a high risk of bias.

### 2.9 Certainty Assessment

To assess the certainty of evidence, the GRADE approach was applied [23]. The assessment considered the risk of bias, inconsistency of results, indirectness of evidence, imprecision, and publication bias. Based on these domains, the overall certainty of evidence was rated as very low, low, moderate, or high.

## 3 Results

### 3.1 Study Selection

**FIGURE 1:**
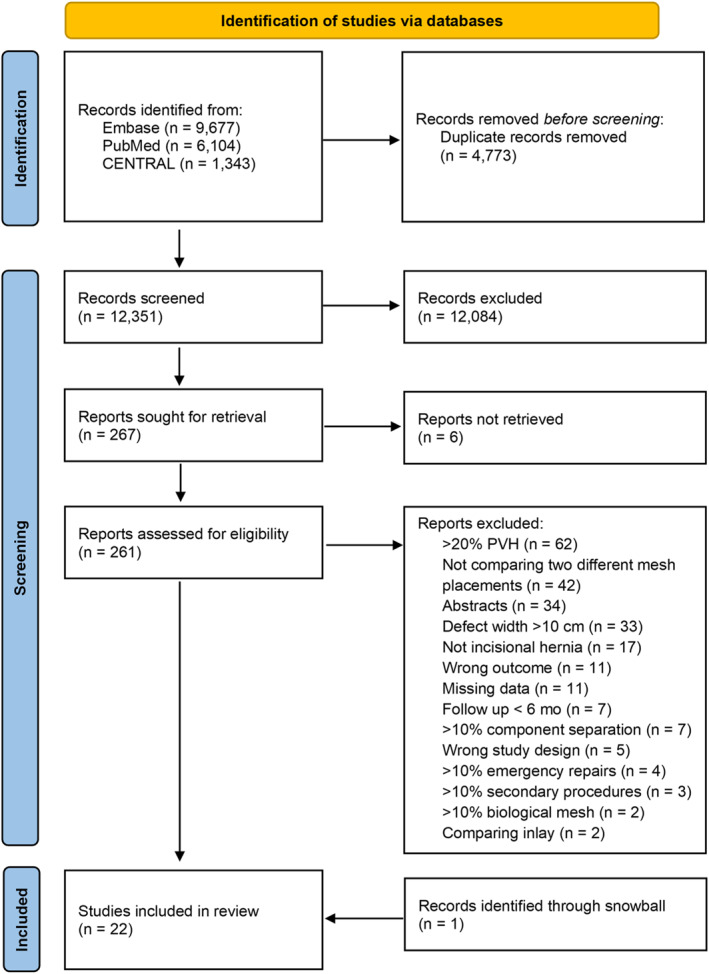
PRISMA 2020 flow diagram. mo, months; *n*, number of reports; PVH, primary ventral hernia.

A total of 21 studies [24, 25, 26, 27, 28, 29, 30, 31, 32, 33, 34, 35, 36, 37, 38, 39, 40, 41, 42, 43, 44] were included through the search strategy, and 1 study [45] was included through backward citations (Figure 1). In total, 20 authors were contacted by e‐mail to obtain additional data or to clarify if studies were eligible for inclusion (Supporting Information S2). Six authors provided additional data, and four studies [30, 35, 40, 45] were included in the review.

### 3.2 Study Characteristics

Six RCTs and 16 cohort studies comprising a total of 10,832 patients were included (Table 1). Among these, 908 patients underwent repair with onlay, 372 with retromuscular, 297 with preperitoneal, 4715 with IPOM, and 4540 with sublay (pooling retromuscular and preperitoneal). No studies pooled primary ventral and incisional hernias. Most studies compared 2 mesh placements, while 1 study [41] compared 3 different placements. All included studies had medium to large hernia defects (3–10 cm); 2 studies [29, 31] limited inclusion to defects larger than 3 cm, and 1 study [38] to defects larger than 4 cm. Most studies [28, 29, 31, 32, 37, 38, 39, 42, 43, 44] compared laparoscopic to open surgery. One study [41] compared mesh placements but included a mixed cohort of laparoscopic and open repair. Six studies [24, 26, 34, 35, 36, 40] exclusively investigated open repairs, and 1 study [33] investigated laparoscopic repairs. Four studies [25, 27, 30, 45] did not specify the surgical approach. Most studies [26, 27, 29, 31, 32, 33, 36, 40, 42, 44] reported a mean or median age between 50 and 60 years old, but 1 study [43] had a younger population (33–36 years old). In most studies [27, 28, 29, 31, 36, 37, 39, 40, 42, 44], recurrence was assessed by clinical examination. One study [30] utilized hospital records to identify recurrences, while another study [43] conducted telephone interviews in which patients reported recurrences. One study [41] reported reoperations extracted from a registry as a proxy for recurrence. Nine studies [24, 25, 26, 32, 33, 34, 35, 38, 45] did not specify how recurrence was assessed.

**TABLE 1:** Study characteristics.

Authors	Comparing	Patients *n*	Follow‐up (mo)	Female *n* (%)	BMI (kg/m^2^)	Diabetes *n*	Smoking *n*	Recurrent repair *n* (%)	Defect width (cm)
RCT									
Bakka 2025 [24]	Onlay	30	18^a^	28 (93)	—	0	0	5 (83)	6.8^a^ (SD 7.3)
	Retromuscular	30	17^a^	27 (90)		0	0		5^a^ (SD 5.4)
Bharti 2024 [25]	Onlay	50	6^c^	20 (40)	27^a^	9	12	0 (0)	5.2^a^ (SD 2.1)
	Preperitoneal	50		18 (36)	28^a^	10	13		5.5^a^ (SD 2.0)
Rawat 2023 [26]	Onlay	30	6^a^	21 (70)	—	6	4	—	< 10 cm^d^
	Retromuscular	30		19 (63)		8	6		
Sevinç 2018 [27]	Onlay	50	36^b^	28 (56)	23^a^	—	—	—	8.9^a^ (SD 8.6)
	Retromuscular	50	38^b^	36 (72)	26^a^				10.4^a^ (SD 9.6)
Rogmark 2016 [28]	Retromuscular	63	12^c^	39 (62)	29^a^	—	17	—	5^b^ (IQR 3–6)
	IPOM	61		35 (57)	29^a^		14		5^b^ (IQR 4–7)
Eker 2011 [29]	Preperitoneal	100	37^a^	41 (41)	29^a^	—	—	41 (21)	5^b^ (IQR 4–10)
	IPOM	94	34^a^	38 (40)	28^a^				5^b^ (IQR 4–8)
Cohorts									
Hassan 2024 [30]	Onlay	50	6^a^	60 (63)	28^a^	—	—	0 (0)	< 10 cm^d^
	Sublay	46			26^a^				
Yavuz 2024 [31]	Onlay	30	21^a^	15 (25)	31^a^	—	—	—	> 3 cm^d^
	IPOM	30	18^a^		31^a^				
Paic 2023 [32]	Onlay	28	12^a^	15 (54)	—	—	—	—	5.3^a^ (SD 2.4)
	IPOM	42		23 (55)					4.9^a^ (SD 1.8)
Taşdelen 2023 [33]	Retromuscular	30	24^a^	22 (73)	32^a^	8	3	—	6^a^ (SD 5.1)
	IPOM+	44	45^a^	30 (68)	34^a^	8	11		5.9^a^ (SD 5.6)
Kumar 2022 [34]	Onlay	41	19^a^	38 (93)	25^a^	2	—	3 (7)	4.3^a^ (SD 0.7)
	Retromuscular	46	16^a^	38 (83)	26^a^	4		5 (11)	4^a^ (SD 1.0)
Al Taha 2020 [35]	Onlay	40	24^c^	29 (73)	< 40	—	0	0 (0)	< 10 cm^d^
	IPOM+	40		22 (55)			0		
Fafaj 2020 [36]	Sublay	3624	36^b^	1808 (50)	32^a^	725	380	1534 (42)	10^a^ (SD 7)
	IPOM (90% IPOM+)	587		297 (51)	32^a^	92	74	176 (30)	5^a^ (SD 5)
Köckerling 2019 [37]	Sublay	3965	12^c^	—	29^a^	—	—	0 (0)	—
	IPOM (24% IPOM+)	3965			30^a^				
Savitha 2018 [45]	Onlay	25	6–24^b^	24 (48)	—	—	—	—	< 10 cm^d^
	Retromuscular	25		23 (46)					
Mohamed 2017 [38]	Retromuscular	31	27^a^	15 (48)	30^a^	—	—	—	8.0^a^ (SD 1.0)
	IPOM	29	27^a^	9 (31)	30^a^				7.8^a^ (SD 1.2)
Asti 2016 [39]	Retromuscular	70	36^a^	30 (43)	28^a^	—	19	0 (0)	8.2^b^
	IPOM	54		29 (54)	29^a^		12		5.9^b^
Saeed 2014 [40]	Onlay	40	24^c^	32 (80)	—	—	—	—	5.1^a^ (SD 1.4)
	Preperitoneal	40		23 (58)					6.7^a^ (SD 3.1)
Helgstrand 2013 [41]	Onlay	454	21^b^	1728 (53)	—	—	—	593 (18)	7^b^ (IQR 4–13)
	Sublay	323							
	IPOM	258							
Kurmann 2010 [42]	Preperitoneal	25	56^b^	4 (13)	27^a^	—	—	0 (0)	—
	IPOM	13	29^b^	3 (23)	29^b^				
Qadri 2010 [43]	Onlay	40	26^a^	30 (75)	29^b^	—	—	—	8.4^a^ (IQR 4.5–12.4)
	IPOM	40	28^a^	28 (70)	29^b^				8.9^a^ (IQR 5.0–13.1)
van't Riet 2002 [44]	Preperitoneal	76	17^a^	36 (47)	29^b^	—	—	20 (26)	7^b^ (IQR 1–30)
	IPOM+	25	15^a^	12 (48)	28^b^			9 (36)	6^b^ (IQR 2–10)

*Note:* Sublay represents pooled retromuscular and preperitoneal mesh placements. —, not reported.

Abbreviations: BMI, body mass index; IQR, interquartile range; mo, months; *n*, number of participants; SD, standard deviation.

^a^ Mean.

^b^ Median.

^c^ Not reported if the value is mean or median.

^d^ Inclusion criteria.

### 3.3 Risk of Bias in Studies

**FIGURE 2:**
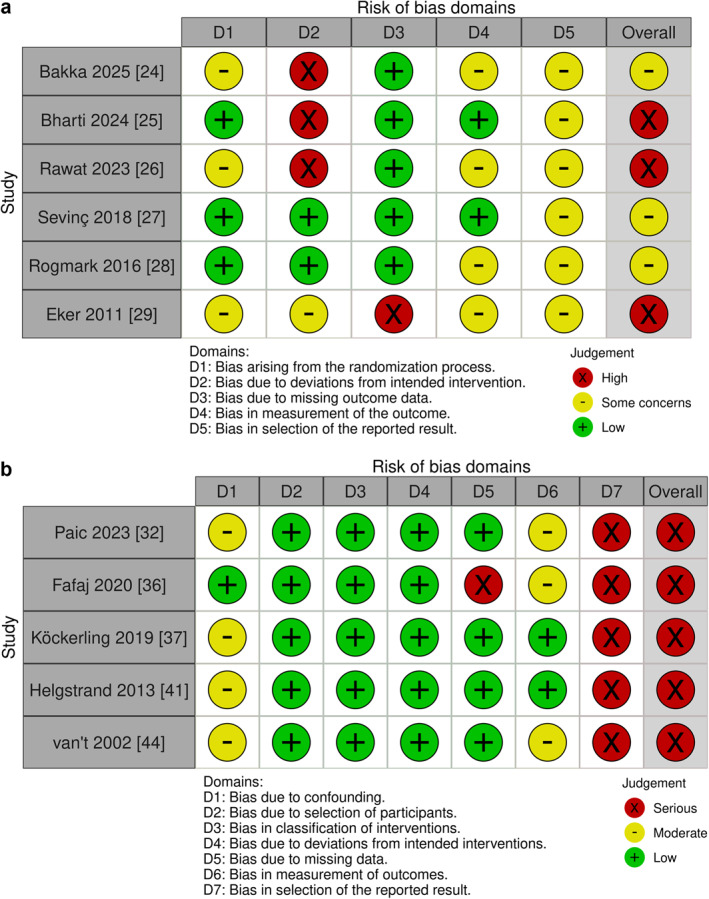
Bias assessment. Cochrane risk‐of‐bias 2 (RoB 2) tool assessment for randomized controlled trials, and Risk Of Bias In Non‐Randomized Studies—of Interventions, version 2 (ROBINS‐I V2). The cohort studies not shown in the figure were rated with critical risk of bias due to uncontrolled confounding and therefore were not proceeded further with ROBINS‐I [17].

The risk of bias for chronic pain outcomes matched that of recurrence, that is, studies with a high risk of bias for recurrence also had a high risk of bias for chronic pain (Figure 2). Most RCTs [24, 25, 26, 27, 28] had a low risk of bias in domain 3 (missing outcome), although one study [29] was rated high risk due to a 25% loss to follow‐up. Three RCTs [24, 25, 26] were rated high risk in domain 2 (deviations from intended intervention) due to limited information regarding adherence to intended intervention groups, and all RCTs were rated with some concerns in domain 5 (selection of the reported results), because they lacked a pre‐specified analysis plan. Overall, three RCTs [24, 27, 28] were rated as having some concerns, and three RCTs [25, 26, 29] were rated high risk.

Eleven cohort studies [30, 31, 33, 34, 35, 38, 39, 40, 42, 43, 45] did not adjust for confounding. According to the guideline, studies with uncontrolled confounders should not undergo further assessment with ROBINS‐I V2 but be rated as having a critical risk of bias [17]. The remaining 5 cohort studies [32, 36, 37, 41, 44] were evaluated using ROBINS‐I V2, and all were rated as having a serious risk of bias in domain 7 (selection of the reported results): mainly because most studies lacked a pre‐specified analysis plan. One study [36] was also rated with a serious risk of bias in domain 5 (missing data), as they did not address missing data for follow‐up. No other studies had a serious risk of bias in the other domains (Figure 2). Overall, a serious risk of bias was present among all the cohort studies.

### 3.4 Recurrence

The highest crude recurrence rates were observed for onlay and preperitoneal placements (11.7% and 12.8%, respectively), while IPOM had a rate of 5.7%, and retromuscular placement had the lowest crude recurrence rate at 3.0% (Table 2). Meta‐analysis was conducted for 5 pairwise comparisons of mesh placements, including all studies except those that pooled retromuscular and preperitoneal placements as sublay. A significant difference was found between retromuscular and onlay placements, favoring retromuscular (RR 3.2, 95% CI 1.1–9.2) (Figure S1). No significant differences were found between other mesh placements compared (Figure S1).

**TABLE 2:** Summary of crude recurrence and risk of bias assessment.

Study	Design	Recurrence *n/N* (%)					Risk of bias
		Onlay	Retromuscular	Preperitoneal	IPOM	Sublay	
Bakka 2025 [24]	RCT	0/30 (0)	0/30 (0)	—	—	—	Some concerns
Bharti 2024 [25]	RCT	4/50 (8)	—	1/50 (2)	—	—	High
Rawat 2023 [26]	RCT	3/30 (10)	0/30 (0)	—	—	—	High
Sevinç 2018 [27]	RCT	3/50 (6)	1/50 (2)	—	—	—	Some concerns
Rogmark 2016 [28]	RCT	—	1/63 (2)	—	5/61 (8)	—	Some concerns
Eker 2011 [29]	RCT	—	—	14/100 (14)	17/94 (18)	—	High
Hassan 2024 [30]	Cohort	2/50 (4)	—	—	—	1/46 (2)	Critical
Yavuz 2024 [31]	Cohort	4/30 (13)	—	—	1/30 (3)	—	Critical
Paic et 2023 [32]	Cohort	3/28 (11)	—	—	0/42 (0)	—	Serious
Taşdelen 2023 [33]	Cohort	—	1/27 (4)	—	2/42 (5)	—	Critical
Kumar 2022 [34]	Cohort	5/41 (12)	2/46 (4)	—	—	—	Critical
Al Taha 2020 [35]	Cohort	3/40 (8)	—	—	0/40 (0)	—	Critical
Fafaj 2020 [36]	Cohort	—	—	—	9/22 (41)	55/206 (27)	Serious
Köckerling 2019 [37]	Cohort	—	—	—	167/3965 (4)	163/3965 (4)	Serious
Savitha 2018 [45]	Cohort	2/25 (8)	0/25 (0)	—	—	—	Critical
Mohamed 2017 [38]	Cohort	—	0/31 (0)	—	1/29 (3)	—	Critical
Asti 2016 [39]	Cohort	—	6/70 (9)	—	4/54 (7)	—	Critical
Saeed 2014 [40]	Cohort	3/40 (7)	—	0/40 (0)	—	—	Critical
Helgstrand 2013 [41]	Cohort	73/454 (16)	—	—	55/258 (11)	39/323 (12)	Serious
Kurmann 2010 [42]	Cohort	—	—	9/31 (29)	2/13 (15)	—	Critical
Qadri 2010 [43]	Cohort	1/40 (3)	—	—	1/40 (3)	—	Critical
van't Riet 2002 [44]	Cohort	—	—	14/76 (18)	4/25 (16)	—	Serious

*Note:* Risk of bias assessment for randomized controlled trials was graded as either low risk, some concerns, or high risk; Risk of bias assessment for cohorts was graded as either low, moderate, serious, or critical risk. —, not reported.

Abbreviation: *n/N*, number of participants with recurrences out of all participants.

**FIGURE 3:**
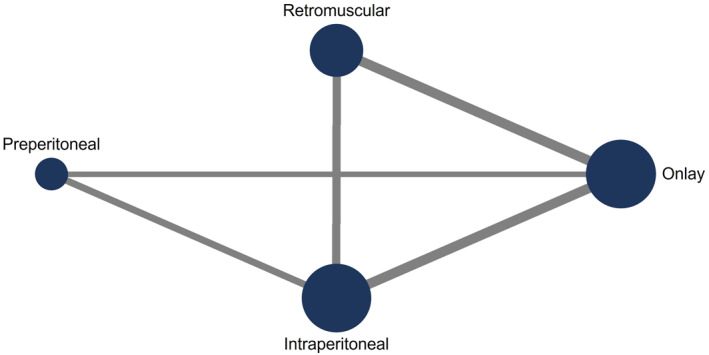
Mesh placements compared in the network meta‐analysis. Each dot represents a mesh placement, and dot size reflects the number of patients contributing data. Lines indicate direct comparisons between placements, with line thickness corresponding to the number of studies informing each comparison.

**FIGURE 4:**
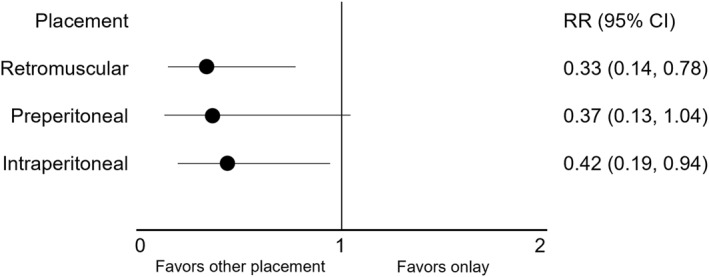
Network meta‐analysis of recurrence rates. Mesh placements are compared with onlay as the reference group using a random‐effects model. Dots represent risk ratio (RR) estimates, and error bars indicate 95% confidence intervals (CI). An RR below one indicates a lower risk of recurrence compared with onlay.

In the main analysis, there were 19 studies [24, 25, 26, 27, 28, 29, 31, 32, 33, 34, 35, 38, 39, 40, 41, 42, 43, 44, 45] included in the network meta‐analysis comparing 4 different mesh placements (Figures 3 and 4). Compared with onlay placement, retromuscular placement was associated with a lower risk of recurrence (RR 0.3, 95% CI 0.1–0.8). IPOM placement was also associated with a lower risk of recurrence as well (RR 0.4, 95% CI 0.2–0.9).

In a sensitivity analysis using the same studies as in the main analysis, but analyzing IPOM with and without defect closure separately, the effect estimates were similar to IPOM in the main analysis for both subgroups, but without statistical significance. When excluding studies using reoperation as a proxy for recurrence [41], all placements were significantly superior to onlay. In the analysis, including only RCTs, no significant difference was found between mesh placements. The remaining sensitivity analyses were neither significant.

In the GRADE assessment, the initial level of certainty was rated as high, as the review included RCTs and cohort studies evaluated with the ROBINS‐I V2 tool. However, the level of certainty was downgraded due to several factors: a general high risk of bias, imprecision reflected by wide confidence intervals in the meta‐analyses, and inconsistency arising from variations in outcomes across studies, with findings differing in both direction and magnitude. Consequently, the overall certainty in the evidence was rated as very low. No factors warranted an upgrade of the evidence level.

### 3.5 Chronic Pain

Two studies [28, 34] assessed chronic pain using a visual analog scale, 1 study [37] conducted interviews at follow‐up, and 1 [39] used the SF‐36 questionnaire (Table 3). The studies were heterogeneous due to various methods of measurement, and the follow‐up ranged from 12 to 36 months. However, 1 study [28] found lower intensity of chronic pain after 12 months with retromuscular compared with IPOM placement.

**TABLE 3:** Chronic pain reporting.

Author	Study design	Method	Chronic pain				*p* value	Risk of bias
			Onlay	Retromuscular	IPOM	Sublay		
Rogmark 2016 [28]	RCT	VAS 0–100, continuous. Mean value.	—	5	6	—	< 0.01	Some concerns
Kumar 2022 [34]	Cohort	VAS 0–10. % of patients with VAS > 1.	10%	4%	—	—	0.320	Critical
Köckerling 2019 [37]	Cohort	Pain when exercising, % of patients saying yes.	—	—	15%	15%	0.796	Serious
Asti 2016 [39]	Cohort	SF‐36* bodily pain 0–100, continuous. Mean value.	—	58	55	—	0.748	Critical

*Note:* The column *method* describe how each study measured and reported pain outcomes. Visual analog scale (VAS) is used to assess pain, ranging from 0 (no symptoms) to 100 (worst imaginable suffering) [28]. Short Form 36 (SF‐36) is a self‐reported measurement of wellbeing, consisting of 36 items with two items focusing on bodily pain. Score ranges from 0–100. Low score is pain, high score is no pain [39, 46]. —, not reported.

## 4 Discussion

This systematic review with network meta‐analysis evaluated the impact of various mesh placements on recurrence rates and chronic pain. We found that retromuscular and intraperitoneal placement had a lower risk of recurrence compared with onlay placement. However, the findings for intraperitoneal mesh were not robust due to inconsistency with the pairwise meta‐analysis and across sensitivity analyses. Chronic pain was reported in only 4 studies, which were too heterogeneous to be pooled in a meta‐analysis. Overall, the included studies were at high risk of bias, and the certainty of evidence was very low.

One review [47] evaluating prophylactic mesh in elective laparotomy found no difference in incidence rates between retromuscular and onlay mesh placements. In contrast, our analysis compared mesh placements in patients with established incisional hernias. We assessed both recurrence and chronic pain, finding that retromuscular placement may lower recurrence rates compared with onlay.

### 4.1 Strengths and Limitations

This review has several strengths. It was conducted in accordance with the PRISMA‐NMA guideline [11], and the protocol was registered at PROSPERO before data extractions to ensure transparency. The search strategy was developed in collaboration with an information specialist, enhancing the likelihood of identifying all relevant literature [48]. We included a broad search string, used forward and backward citation searches [13], and had no language restrictions to ensure a larger coverage. Unlike previous research [3], this review was limited to incisional hernia and did not pool primary ventral and incisional hernias, thereby reducing the clinical heterogeneity [1]. Another strength was the use of network meta‐analysis, enabling comparison of more than 2 mesh placements by combining both direct and indirect comparisons, thus increasing the sample size for more certain estimates.

Several limitations of this review should be acknowledged. Six potentially eligible studies could not be retrieved, and if these studies differed from those of the included studies, this could influence the overall results. Cohort studies were included and constituted the majority of the evidence base. However, cohort studies introduce potential confounding and selection bias due to lack of randomization, as surgeons may choose the mesh placement, they consider most suitable for their patients [49]. When cohort studies were excluded in the sensitivity analysis, no significant differences were observed between the mesh placements. However, this analysis was based on a limited number of RCTs and a substantially smaller sample size, resulting in reduced statistical power. One study [41] used reoperation as a proxy for recurrence, which likely underestimates the true recurrence rate [21], but this was also investigated in the sensitivity analysis, which had similar findings to the network meta‐analysis. This review did not consider the impact of mesh fixation techniques, as their influence on recurrence remains unclear. For example, 1 study [50] suggested that suture as mesh fixation may lower recurrence for IPOM. Besides excluding Physiomesh, resorbable, and biological mesh [51], mesh types and materials were not taken into consideration, which could also affect the risk of recurrence [52]. Limitations were also present in the included studies. Significant clinical and methodological heterogeneity was observed, reflected in broad confidence intervals in the meta‐analyses, and many studies did not adjust for potential confounders, leading to critical or high risk of bias. Variations in surgical approach and patient selection may also challenge transitivity and affect indirect comparisons beyond mesh placement. Another limitation is that the preperitoneal group included open repairs. As laparoscopic techniques are increasingly adopted [53], the extent to which these findings can be extrapolated to laparoscopic preperitoneal repair remains uncertain. Notably, 1 study [27] employed onlay mesh placement for incisional hernias with a mean defect width of 8.9 cm, which is uncommon for large incisional hernias [8].

### 4.2 Perspectives

Current guidelines recommend retromuscular mesh placement [6], which aligns with our findings of this review. However, we also found that IPOM placement may also reduce recurrence rates. There has been a decline in the use of IPOM, and one possible explanation for its lower popularity among surgeons is the perception that it poses a risk of serious complications, such as bowel adhesions and obstruction, although this concern is supported by very limited evidence, highlighting the need for further research [49]. For most outcomes, current evidence remains limited due to the pooling of incisional and primary ventral hernias in studies [1], an inadequate number of RCTs, and high risk of bias, resulting in very low certainty. Another important point is that all the included studies had medium and large hernias; therefore, the findings cannot be generalized to small hernias. These results underscore the need for further research, and we therefore suggest conducting more RCTs and high‐quality cohort studies that only include patients with incisional hernias. A limited number of studies [28, 34, 37, 39] reported chronic pain, and the outcome was assessed using heterogeneous definitions and measurements, precluding quantitative synthesis and limiting comparability across studies. This inconsistency reflects a broader gap in hernia research, in which patient‐reported outcomes are often underreported [54]. One publication [55] discussed that the clinical measurements, such as recurrence and readmission, do not necessarily correspond with the level of patient satisfaction. Given that hernia repair ultimately aims to improve patients' quality of life [55], future studies should integrate standardized patient‐reported outcomes (PROs) to ensure that clinical success aligns with patient satisfaction and functional recovery.

## 5 Conclusion

Retromuscular mesh placement may reduce recurrence rates in patients with medium to large incisional hernias compared with onlay mesh. Although intraperitoneal mesh also lowered the risk of recurrence, the findings were not robust across analyses. The results should be interpreted with caution, as the certainty of the current evidence is very low due to high risk of bias and clinical and methodological heterogeneity. There is insufficient evidence to draw conclusions regarding the impact of mesh placement on chronic pain following incisional hernia repair. Future studies, focusing on incisional hernias and with a low risk of bias, are warranted to confirm these findings.

## Author Contributions

**Camilla Witthøft:** writing – original draft, conceptualization, methodology, formal analysis, investigation, supervision, resources, visualization, project administration. **Usamah Ahmed:** writing – review and editing, investigation, supervision, conceptualization, methodology, project administration. **Evy Á Lakjuni Guttesen:** writing – review and editing, investigation, conceptualization, methodology. **Jacob Rosenberg:** writing – Review and editing, supervision, conceptualization, methodology, project administration. **Jason Joe Baker:** writing – review and editing, investigation, supervision, conceptualization, methodology, project administration.

## Funding

The authors have nothing to report.

## Conflicts of Interest

The authors declare no conflicts of interest.

## Supporting information


**Figure S1:** Meta‐analyses.

Supporting Information S2

## Data Availability

The data that support the findings of this study are available from the corresponding author upon reasonable request.
