# Supplementary material for: Association Between Mesh Placement and Recurrence and Chronic Pain After Incisional Hernia Repair: A Systematic Review and Network Meta‐Analysis
Source: World J Surg. 2026 May 6;50(6):1545–56. doi: 10.1002/wjs.70390 (PMC13242064; doi:10.1002/wjs.70390)
Supplement: Supplementary file 1 — Figure S1: Meta‐analyses. [file WJS-50-1545-s002.docx]

**Title:** Retromuscular mesh placement may lower recurrence in incisional hernia repair: a systematic review and network meta-analysis

**Journal:** World Journal of Surgery

**Authors:** Camilla Witthøft, Usamah Ahmed, Evy Á Lakjuni, Jacob Rosenberg, Jason Joe Baker.

Center for Perioperative Optimization, Department of Surgery, Copenhagen University Hospital - Herlev and Gentofte, Borgmester Ib Juuls Vej 1, DK-2730 Herlev, Denmark

**Corresponding author:** Camilla Witthøft, e-mail: [camillawitthoft@outlook.dk](mailto:camillawitthoft@outlook.dk)

**Figure 1** Meta-analyses


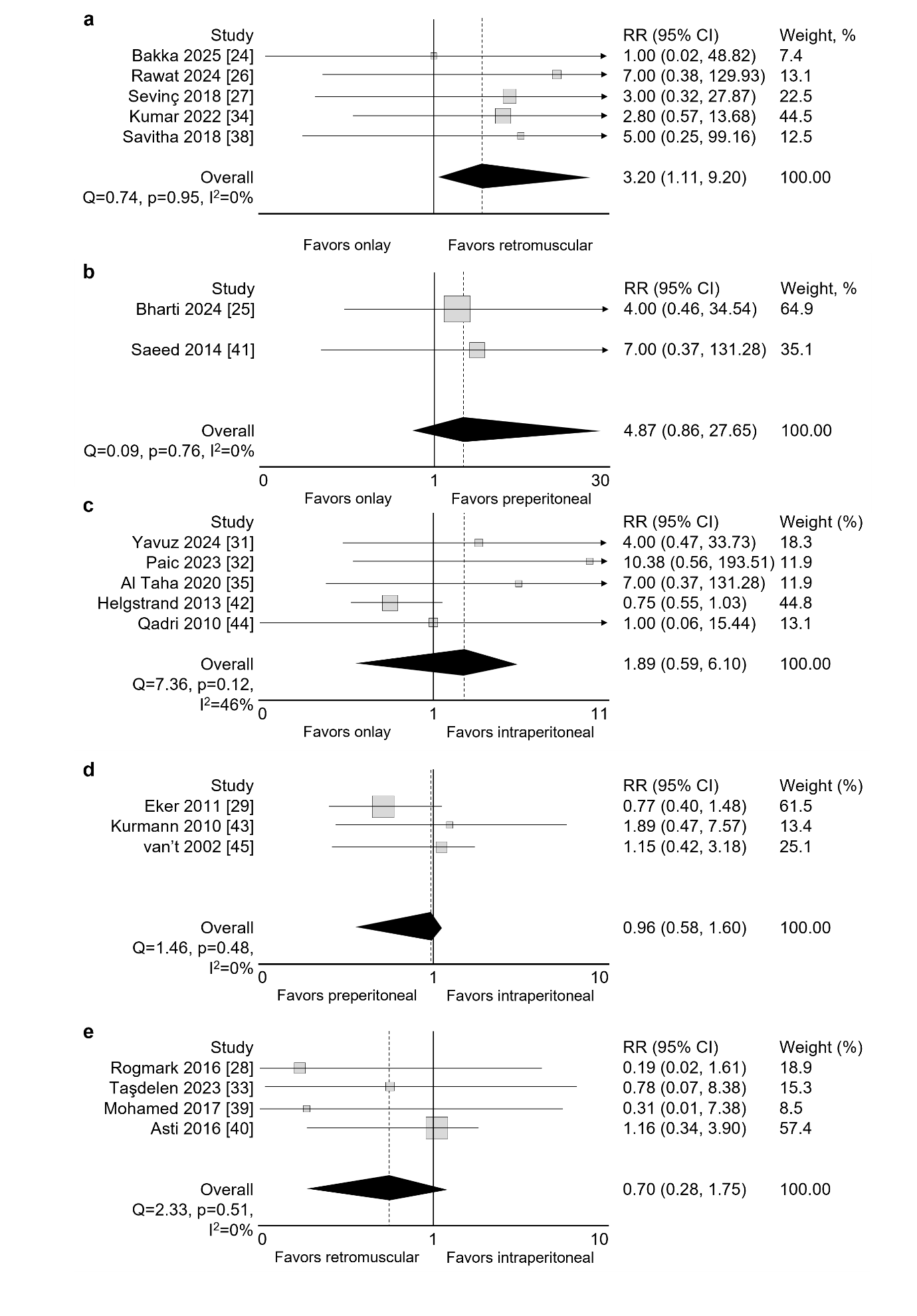


a: Onlay compared with retromuscular placement. b: Onlay compared with preperitoneal placement. c: Onlay compared with intraperitoneal placement. d: Preperitoneal compared with intraperitoneal placement. e: Retromuscular compared with intraperitoneal placement. Random-effect model was used. The square represents the estimate of the risk ratio, the error bars indicate 95% confidence intervals, the vertical line represents RR = 1, and the diamond represents the overall effect. RR, risk ratio; CI, confidence intervals.
