# Supplementary material for: Association Between Mesh Placement and Recurrence and Chronic Pain After Incisional Hernia Repair: A Systematic Review and Network Meta‐Analysis
Source: World J Surg. 2026 May 6;50(6):1545–56. doi: 10.1002/wjs.70390 (PMC13242064; doi:10.1002/wjs.70390)
Supplement: Supplementary file 2 — Supporting Information S2 [file WJS-50-1545-s001.docx]

**Title:** Association between mesh placement and recurrence and chronic pain after incisional hernia repair: a systematic review and network meta-analysis

**Journal:** World Journal of Surgery

**Authors:** Camilla Witthøft, Usamah Ahmed, Evy Á Lakjuni Guttesen, Jacob Rosenberg, Jason Joe Baker.

Center for Perioperative Optimization, Department of Surgery, Copenhagen University Hospital - Herlev and Gentofte, Borgmester Ib Juuls Vej 1, DK-2730 Herlev, Denmark

**Corresponding author:** Camilla Witthøft, e-mail: [camillawitthoft@outlook.dk](mailto:camillawitthoft@outlook.dk)

**Excluded studies**

## Six studies [1–5] could not be retrieved for full-text screening and were therefore excluded. Studies [6–9] that could potentially be included, but lacked essential data, and for which no e-mail for the author was available, were also excluded. Twenty authors were contacted by e-mail to obtain or to clarify if studies were eligible for inclusion, 6 authors provided additional data, for which four studies were included in the review. Two studies [10,11] were excluded, as they did not meet the inclusion criteria. One author [12] reported that the requested data were unavailable. Of the 13 authors who did not respond, 10 studies [13–22] were excluded due to missing data, and three studies [23–25] were retained, as the additional data were not critical for inclusion.

## **References:**

[1] L. Napolitano, N. Di Bartolomeo, L. Aceto, M. Waku, P. Innocenti, “Use of prosthetic materials in incisional hernias: our clinical experience,” *G Chir* **25**, no. 4 (2004): 141–145.

[2] S. C. P. Katti, M. J. Mani, B. P. Nagula, “Comparative study of pre-peritoneal versus onlay mesh repair of ventral hernias,” *J Cardiovasc Dis Res* **14**, no. 8 (2023): 2159–2165.

[3] S. K. Garg, S. Kumar, “A comparative evaluation of management of incisional hernia,” *Eur J Mol Clin Med* **9**, no. 1 (2022): 1156–1160.

[4] Z. M. Demetrashvili, R. D. Magalashvili, G. V. Lobzhanidze, K. R. Khutsishvili, L. Z. Labauri, “Experience in treatment of postoperative ventral hernias,” *Khirurgiia* **11** (2008): 44–46.

[5] G. Battistini, M. D’Amato, M. Abete, et al., “Large eventrations: personal series, retrospective analysis and proposal of onlay’s technique,” *Chirurgia* **24**, no. 2 (2011): 61–65.

[6] C. D. K. Raja, V. M. Rao, C. Satyanarayana, “Open versus laparoscopic ventral hernia repair: a randomized clinical trial,” *IJPCR* **16**, no. 6 (2024): 69–72.

[7] C. S. Naik, N. Ahmed, M. S. Aishwarya, S. M. Akram, “Outcomes of two specific surgical methods for repairing incisional hernias: open retromuscular mesh placement (sublay) and onlay mesh placement,” *Res J Med Sci* **18**, no. 11 (2024): 410–412, https://doi.org/10.36478/10.36478/makrjms.2024.11.410.412.

[8] R. Ahmad, J. Bharti, “Comparison and future prospects of ventral hernia repair: laparoscopic vs. open techniques,” *IJPCR* **16**, no. 5 (2024): 1303–1306.

[9] S. Kumar, “Onlay and sublay mesh repair in incisional hernias: randomized comparative study,” *IJPCR* **13**, no. 5 (2021): 518–523.

[10] M. Kumar, N. Chandra, “Outcomes of open retrorectus versus laparoscopic intraperitoneal onlay mesh repair for ventral hernias patients: a comparative study,” *Int J Life Sci Biotechnol Pharma Res* **11**, no. 1 (2022): 489–495.

[11] C. C. Petro, S. M. Maskal, D. B. Renton, et al., “Robotic enhanced-view totally extraperitoneal vs intraperitoneal onlay mesh evaluation: 1-year exploratory outcomes of the REVEAL randomized clinical trial,” *J Am Coll Surg* **237**, no. 4 (2023): 614–620, https://doi.org/10.1097/xcs.0000000000000784.

[12] V. Kumar, G. Rodrigues, C. Ravi, S. Kumar, “A comparative analysis on various techniques of incisional hernia repair-experience from a tertiary care teaching hospital in South India,” *Indian J Surg* **75**, no. 4 (2013): 271–273, https://doi.org/10.1007/s12262-012-0644-z.

[13] N. P. Suthar, V. M. Patel, M. Trivedi, “Incisional hernia: predictive factors, clinical presentation and management,” *IJTPR* **14**, no. 12 (2024): 167–170.

[14] P. P. Binthaf, G. Parag, “A comparative study between ETEP vs. IPOM repair for ventral hernia,” *Hernia* **29**, no. 1 (2025): 88, https://doi.org/10.1007/s10029-025-03280-4.

[15] S. H. Gondal, I. H. Anjum, “Sutureless sublay verses onlay mesh hernioplsty in incisional hernia repair: a comparative study at teaching hospital, Lahore,” *Pak J Med Health Sci* **6**, no. 1 (2012): 238–241.

[16] M. R. Behera, “A comparative analysis of open and laparoscopic ventral hernia repair techniques,” *J Cardiovasc Dis Res* **14**, no. 9 (2023): 1433–1442.

[17] N. Nagoti, N. Dwarakanath, P. P. Varma, “A comparative study on laparoscopic ETEP vs laparoscopic IPOM plus for ventral hernia,” *Int J Med Pub Health* **14**, no. 4 (2024): 953–957, https://doi.org/10.70034/ijmedph.2024.4.175.

[18] S. Natarajan, S. Meenaa, K. A. Thimmaiah, “A randomised prospective study to evaluate preperitoneal mesh repair versus onlay mesh repair and laparoscopic IPOM in incisional hernia surgery,” *Indian J Surg* **79**, no. 2 (2017): 96–100, https://doi.org/10.1007/s12262-015-1430-5.

[19] M. Coskun, Y. Peker, F. Tatar, N. Cin, H. Kar, M. C. Kahya, “Median incisional hernia and the factors affecting the recurrence of median incisional hernia repair,” *J Clin Pract Res* **31**, no. 3 (2009): 244–249.

[20] B. T. Fry, R. A. Howard, J. R. Thumma, E. C. Norton, J. B. Dimick, K. H. Sheetz, “Surgical approach and long-term recurrence after ventral hernia repair,” *JAMA Surg* **159**, no. 9 (2024): 1019–1028, https://doi.org/10.1001/jamasurg.2024.1696.

[21] B. J. Ramshaw, P. Esartia, J. Schwab, et al., “Comparison of laparoscopic and open ventral herniorrhaphy,” *Am Surg* **65**, no. 9 (1999): 827–832.

[22] M. J. Shakir, I. Sadiq, N. Khan, M. A. Ahmed, S. S. Ashfaq, A. Ijaz, “Laparoscopic ventral hernia repair versus open hernioplasty,” *Pak J Med Health Sci* **15**, no. 10 (2021): 2602–2603, https://doi.org/10.53350/pjmhs2115102602.

[23] R. Yavuz, F. Sağlam, “The comparison of laporoscopic and open procedures in ventral-incisional hernia repair,” *Ann Clin Anal Med* **15**, no. 5 (2024): 364–368, http://doi.org/10.4328/ACAM.22170.

[24] F. Köckerling, T. Simon, D. Adolf, et al., “Laparoscopic IPOM versus open sublay technique for elective incisional hernia repair: a registry-based, propensity score-matched comparison of 9907 patients,” *Surg Endosc* **33**, no. 10 (2019): 3361–3369, https://doi.org/10.1007/s00464-018-06629-2.

[25] A. Kurmann, G. Beldi, S. A. Vorburger, C. A. Seiler, D. Candinas, “Laparoscopic incisional hernia repair is feasible and safe after liver transplantation,” *Surg Endosc* **24**, no. 6 (2010): 1451–1455, https://doi.org/10.1007/s00464-009-0799-2.
